# A Rare Case of Lymphoproliferative Disorder in Pregnancy: A Case Report

**DOI:** 10.7759/cureus.69893

**Published:** 2024-09-22

**Authors:** Priyankha Ramamoorthy, Nagarajan Priyathersini, Divya M, Muthulakshmi Duraisamy

**Affiliations:** 1 Department of Pathology, Sri Ramachandra Institute of Higher Education and Research, Chennai, IND; 2 Department of Obstetrics and Gynecology, Sri Ramachandra Institute of Higher Education and Research, Chennai, IND

**Keywords:** castleman’s disease, frozen, lymph node pathology, pregnancy, retroperitoneum

## Abstract

Lymphoproliferative disorders in pregnancy are rare. Among that, Castleman disease is an uncommon lymphoproliferative disorder in pregnancy with an unknown etiology. Many cases have not been encountered more often in practice and are easily misdiagnosed due to their rarity. We present a case of a 36-year-old pregnant woman with a left para-ovarian mass incidentally diagnosed during the elective caesarean section as a benign lymphoproliferative disorder, sent in frozen. Diagnosing Castleman disease in frozen histopathology is quite challenging for pathologists, and few cases have been reported.

## Introduction

Castleman disease (CD) is a rare non-neoplastic disease of the lymph node. It is a benign condition. Though various theories have been proposed, the proper known cause of the disease remains uncertain and has a female preponderance. Though Castleman disease can occur in various sites, its occurrence in retroperitoneum is unusual and rare.

Here, we report a case of a 36-year-old pregnant female who is taken up for a lower segment cesarean procedure, and during the surgery, a retroperitoneal lesion is identified, which on histopathological evaluation reveals the presence of Castleman disease.

## Case presentation

**Figure 1:**
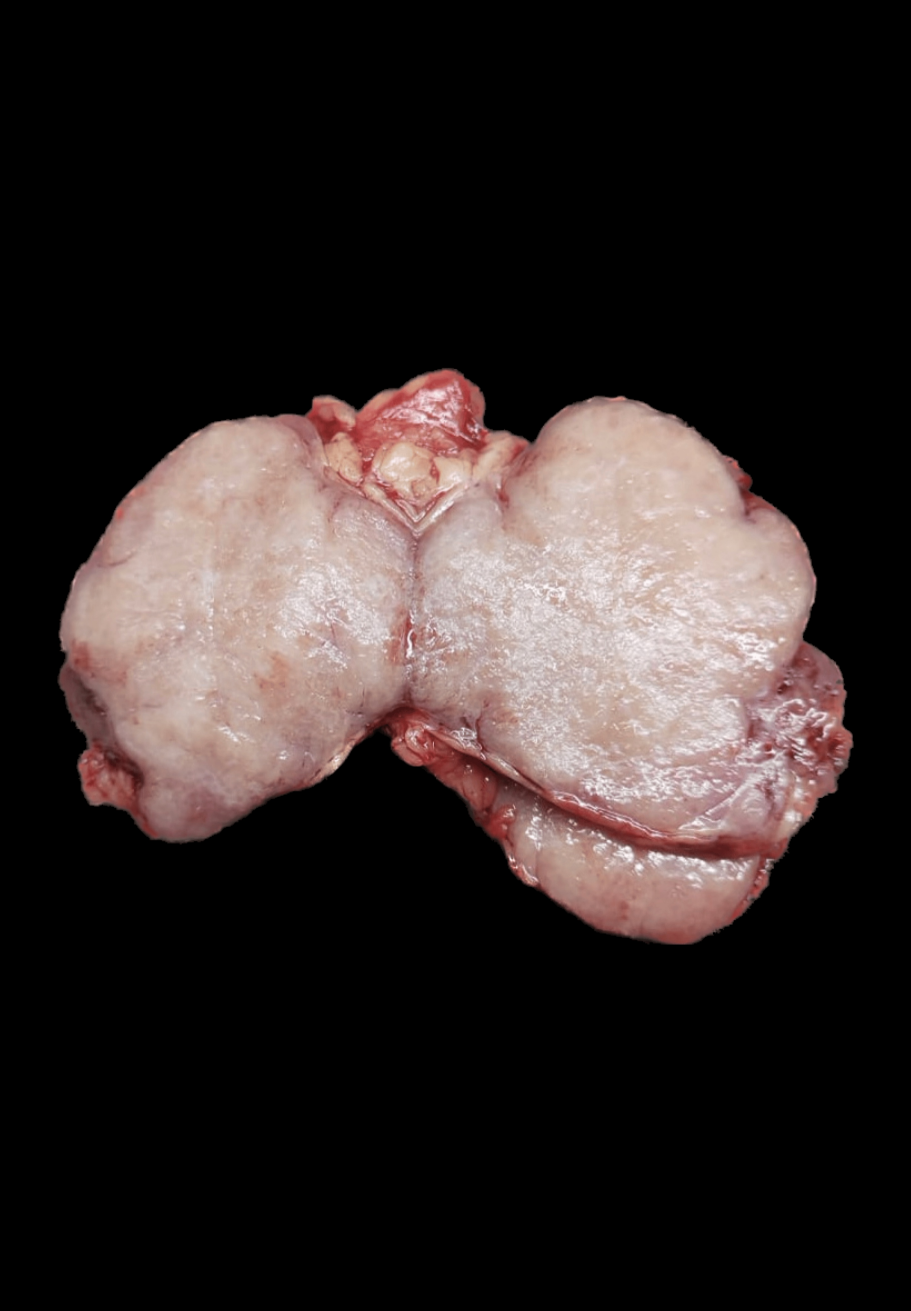
Gross image of the excision specimen of retroperitoneal mass A single grey-white to grey-pink globular mass measuring 8*6*3.5cm

A 36-year-old female who is para 2 and lives 2, 36 weeks and 6 days pregnant is admitted to the hospital for pain in the in the abdomen and safe confinement. She is a known case of diabetes mellitus and is on Metformin 850 mg and injection insulin aspart 20-25-12 units. She attained menarche at the age of 14. Her previous pregnancy 5 years ago was uneventful. Past medical history and laboratory values are unremarkable. Her current pregnancy is uneventful except for her overt diabetes. An elective caesarean section is advised, and unfortunately, due to rupture of membranes and in view of fetal distress, the patient is taken up for surgery. Intraoperatively, the surgeon identifies a solid mass measuring 6x5 cm in the left lateral pelvic wall of the retroperitoneum. Surgical resection of the mass is done and sent for frozen section evaluation (Figure 1). The frozen section on microscopy reveals a lymphoproliferative disorder with atretic germinal centers and is diagnosed as a benign lymphoproliferative disorder. The surgeon, after confirming a benign diagnosis on frozen, went ahead with the delivery of a healthy male baby weighing 3.5 kilograms by performing a lower segment cesarian section.

**Figure 2:**
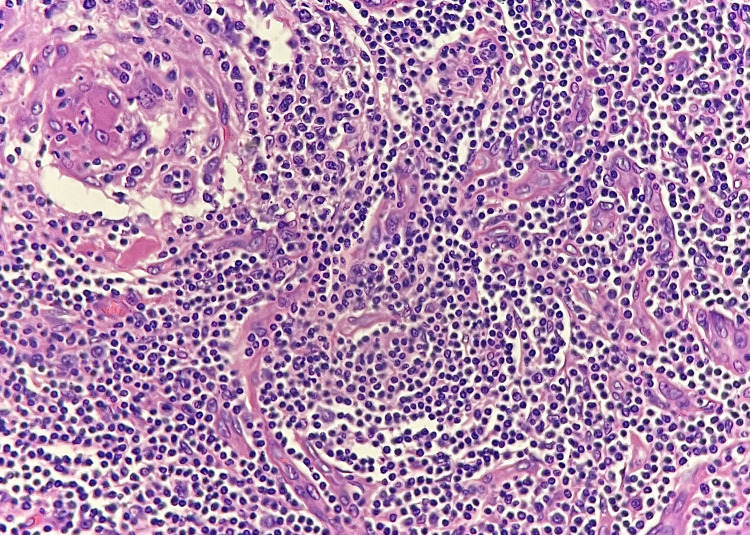
Microscopy image of the retroperitoneal mass 400x H&E lymphoreticular tissue composed of atretic germinal centers traversed by sclerotic penetrating vessels forming lollipop follicles

Routine processing of the specimen shows a well-circumscribed lymphoid tissue with atretic germinal centers traversed by sclerotic penetrating vessels (Figure 2). The differential diagnosis of Castleman’s disease is considered. However, for confirmation and to rule out lymphoma, immunohistochemistry (IHC) evaluation with CD 45, CD20, CD3, CD10, and Ki67 is performed. CD45 shows diffuse positivity, CD10 shows positivity in germinal centers, CD20 shows positivity in B lymphocytes, and CD3 shows positivity in T lymphocytes. This immunohistochemistry pattern suggests a mixed population of T and B lymphocytes. The final pathological diagnosis is given as angiofollicular disease (Castleman’s disease). Her postoperative course is uneventful. 

## Discussion

Castleman disease, initially described in 1956 by Castleman and colleagues, is first identified as a distinct entity characterized by an enlarged lymph node resembling a thymoma located in the anterior mediastinum. This specific presentation is later termed "mediastinal lymph node hyperplasia." However, further research revealed a broader spectrum of the disease, encompassing various types and presentations beyond the initial description. The exact cause of Castleman's disease remains elusive, shrouded in medical mystery. However, several intriguing theories attempt to shed light on its development. One prominent hypothesis proposes an atypical overgrowth of lymphatic tissue triggered by inflammation. While various inflammatory stimuli might play a role, the reasons behind this exaggerated response are still being unraveled [1,2].

Another possibility paints Castleman's disease as a hamartoma, a benign tissue malformation present from birth. However, more research is needed to confirm this link and understand the underlying mechanisms at play. It's crucial to remember that these are just potential explanations, and the true cause likely involves a complex interplay of various factors.

Interestingly, the disease most frequently strikes between the ages of 10 and 30, suggesting a possible link to developmental or immune system processes during that period. However, further investigation [3-5] is necessary to understand this age predilection fully. While historical reports like the one by Testas study [6] suggested a possible female predominance in Castleman's disease, more recent and comprehensive studies haven't identified a clear sex predilection. This means the disease appears to affect individuals of both sexes roughly equally. The common sites of Castleman’s disease are the mediastinum, neck, or axilla. It is very rare in retroperitoneal space, abdominal cavity, pelvis minor, subcutaneous tissue, salivary gland, or muscles. While Castleman disease commonly presents in the mediastinum, neck, or axilla, its occurrence in the retroperitoneum, as encountered in our case, represents a fascinating and infrequent clinical finding, enriching the existing medical literature on this condition. It's worth noting that this disease manifests in three distinct subtypes [4]: hyaline vascular, plasma cell, and a mixed type combining features of both hyaline vascular and plasma cell. This classification system plays a crucial role in guiding diagnosis and treatment approaches. Castleman disease occurs in various subtypes with distinct characteristics. In the context of the retroperitoneum, Kawamura et al. reported the hyaline vascular type, accounting for about 87% of cases. The hyaline vascular subtype can be distinguished by prominent follicular dendritic cells, abnormal cell growth (dysplasia), and an abundance of blood vessels within the lymph node tissue, whereas the plasma cell type exhibits an increased number of follicles harboring enlarged and overactive germinal centers, and only 8% of retroperitoneal cases have been reported. These germinal centers are responsible for producing immune cells. Finally, the mixed type combines both features of both hyaline vascular and plasma cell subtypes, representing 5% of cases forming a unique pathological entity [7,8].

Understanding these distinct subtypes is important for accurate diagnosis and to perform treatment strategies in retroperitoneal Castleman disease. Only limited research exists on Castleman’s disease that has been detected during pregnancy. Pelvic involvement of the disease is very uncommon and can pose diagnostic challenges [7] due to its overlapping features with other pelvic masses. Chemotherapy and immunomodulatory therapies can be preferred for relapse cases [9].

Therefore, careful consideration of Castleman’s Disease in the differential diagnosis is very essential, particularly given the high prevalence (80%) of malignancy among primary retroperitoneal masses. Surgical excision and pathological interpretation remain crucial for definitive diagnosis and tailored patient management.

The diagnosis of Castleman disease is always a challenge in clinical experience since it does not have specific features that could be distinguished from other diseases causing lymphadenopathies [10].

## Conclusions

Castleman disease presents a complex series of challenges during pregnancy. The risk of miscarriages is increased and is accompanied by stress. Further the exclusion of conventional therapies like chemotherapy, radiotherapy, immunotherapy, and drugs are hazardous to the fetus. Additionally, surgical removal of the enlarged mass is complicated in pregnant women due to increased vascularization, which raises the risk of bleeding. Despite this, surgery remains the preferred treatment during pregnancy due to its effectiveness in controlling the disease. While radiation and chemotherapy can sometimes reduce the inoperable tumors for later surgery, these approaches are strictly contraindicated during pregnancy. Therefore, surgical resection remains the choice of treatment for all types of Castleman disease, regardless of pregnancy status. This approach is crucial for both definitive diagnosis and optimal disease management.

In conclusion, Castleman disease can present as a pelvic or retroperitoneal mass and it should be included in the differential diagnoses for such cases. While the specific context of pregnancy adds complexity, complete surgical resection, when possible, remains a highly effective approach for managing Castleman disease in the long term.
